# Identifying clusters of raters with a common notion of diagnosing erosive tooth wear: a step towards improving the accuracy of diagnostic procedures

**DOI:** 10.1186/s40001-024-02260-1

**Published:** 2025-01-09

**Authors:** Kirstin Vach, Carolina Ganss, Nadine Schlueter, Werner Vach

**Affiliations:** 1 Department of Conservative Dentistry, Periodontology and Preventive Dentistry, Hannover Medical School, OE 7740, Carl-Neuberg-Str. 1, 30625 Hannover, Germany; 2 Department of Operative Dentistry, Endodontics, and Paediatric Dentistry, Section of Cariology, Medical Centre of Dentistry, Philipps-University Marburg, Georg-Voigt-Str. 3, 35039 Marburg, Germany; 3 Basel Academy for Quality and Research in Medicine, Steinenring 6, 4051 Basel, Switzerland; 4 Department of Sport Science and Clinical Biomechanics, University of Southern Denmark, Campusvej 55, 5230 Odense M, Denmark

**Keywords:** Multiple raters, Positive rate, Experience, Caries diagnosis, Dentistry, Erosive tooth wear

## Abstract

**Background:**

Heterogeneous results are to be expected when multiple raters diagnose whether the dentine of a tooth with erosive tooth wear (ETW) is exposed or not. Identification of notions (fundamental concepts and understanding) about the diagnostic problem shared by groups of raters can be helpful to develop guidelines and to optimize teaching and calibration procedures. We aim to illustrate how clusters of raters with a common notion can be identified and how first insights about the notions can be obtained.

**Methods:**

This investigation is based on a former study in which 49 tooth surfaces affected by ETW were rated visually by 61 raters (23 scientists, 18 university dentists, 20 dental students) in terms of dentine exposed or not. The true status was determined histologically. Gender, age, professional experience, and specialization of the raters were documented. An algorithm was used to search for clusters of raters with high agreement in their ratings suggesting a common notion. The clusters identified were examined with respect to various aspects.

**Results:**

Four clusters of raters with high agreement could be found. The ratings of the raters in the cluster with the lowest diagnostic accuracy showed the highest correlation with the degree of tissue loss and the background tooth color, whereas the correlation with tissue loss was least in the cluster with highest diagnostic accuracy. The 15 raters of the latter cluster covered both students and dentists with or without specialization in erosion/cariology and/or long experience. This suggests that similar conceptual understanding of ETW can exist independent of professional experience.

**Conclusions:**

The described methodology is useful to identify clusters of raters with a common notion about a specific diagnostic problem. The cluster-specific notions can be further examined based on existing study data or by group-based interviews of the raters of a cluster. This methodology allows investigators to learn more about useful or useless cues in diagnostic decision-making. This information can facilitate development or enhancement of guidelines on diagnostic decision-making.

**Supplementary Information:**

The online version contains supplementary material available at 10.1186/s40001-024-02260-1.

## Introduction

### Why notions are of interest in diagnostic research

Diagnostic procedures are the cornerstone of treatment decisions in all areas of medicine and dentistry [1–3] and are part of the daily practice of healthcare. Whilst some diagnostic questions are clear-cut, for example, the diagnosis of hypertension by measuring blood pressure, others are not. Such difficult diagnoses include, as an example from dentistry, the question of whether or not dentine is exposed in a lesion associated with erosive tooth wear, which is part of many index systems for determining the severity of such lesions [4]. Especially in these difficult diagnostic scenarios, the question of the accuracy of a diagnostic procedure arises.

The accuracy of a diagnostic test is not a constant value, it may vary across different subgroups of patients [5]. For example, old age and the presence of comorbidities may make a diagnosis more difficult compared with young age and presence of only one specific health problem. The accuracy of a test is also typically higher when applied to patients approaching the health care system for the first time than when applied to patients already looking for a diagnosis for some time [6, 7]. Diagnostic accuracy may also depend on the subject (rater) performing the test, in particular on her or his experience or education [5]. Differences between raters are in particular likely if there are multiple signs and symptoms, which can be taken into account, and raters may have different strategies in using these signs and in combining them into a final test decision [8]. Different strategies may reflect differences in the notion of the raters about the diagnostic problem of interest. The term “notion” refers here to a fundamental concept, idea or understanding that forms the base for approaching the diagnostic problem.

Identifying notions shared by subgroups of raters which are associated with a high diagnostic accuracy may help to educate future raters, to inform diagnostic guidelines and to improve the average diagnostic accuracy in the long run.

### How notions can be identified in a diagnostic accuracy study with multiple raters

If raters can vary in their diagnostic notions, it is not wise to include only one rater in a diagnostic accuracy study, as the accuracy observed will depend on the rater chosen. To obtain an estimate of the accuracy we can expect on average in a specific clinical setting, it is necessary to include a sample of raters, representative for this setting. In addition, it becomes possible to search systematically for raters sharing a common notion.

If the signs and symptoms used as cues by the raters are measurable, then it is possible to try to reconstruct the notions of each rater by relating the available signs and symptoms to her or his decisions. For example, this can result in a diagnostic score for each rater giving weights to the single signs and symptoms, and variation in weights across raters allow identifying different notions. Statistical techniques such as latent class analyses with class specific regression coefficients allow identifying such common notions even if the sample size is too small to reconstruct individual notions.

If the signs and symptoms used as cues are not measurable, it might be still possible to identify clusters of raters with a common notion: raters with the same notion should come to very similar decisions. Hence studying the similarity in decisions provides a reasonable starting point.

### The challenge of diagnosing erosive tooth wear

The present investigation was conducted using a data set derived from a previous study [9] that aimed to answer the question of whether the diagnosis of exposed dentine is reliably possible on occlusal/incisal lesions attributed to erosive tooth wear. This form of tooth tissue loss is caused by the effects of chemical and mechanical impacts that naturally affect the teeth over their lifetime. These include acids, for example from food and drinks, and abrasive substances, e.g., in toothpastes, which can lead to the erosion of tooth structure when brushing teeth. This loss of tooth structure is physiological to a certain extent, but excessive consumption of acidic drinks, for example, can lead to pathological wear that can impair the functionality of the teeth. Erosive tooth wear manifests itself in a layer-by-layer removal of hard tooth substance and thus in a loss of tooth contour [10]. Over time, this can lead to the loss of the enamel that covers the crown of the tooth, exposing the underlying dentine.

Dentine has a fundamentally different histological structure to enamel, which is characterised by a lower degree of mineralisation, a higher water content and the presence of a high proportion of collagen. As a result, dentine has a significantly lower microhardness than enamel, which suggests a higher wear rate, and erosion leads to different histological structures in enamel than in dentine [11]. Thus, some erosion-inhibiting agents may be less effective in dentine than in enamel [12]. Both can influence the progression of erosive tooth wear and thus the treatment options. For this reason, the clinical diagnosis of exposed dentine is relevant for treatment planning [13–16]. Finally, many index systems use the “exposed dentine yes/no” criterion as a quantifiable measure of the severity of erosive tooth wear [4].

To date, a number of methods have been proposed for the clinical monitoring of the progression of erosive tooth wear, including various indices as well as the superimposition of intraoral scans [17] or other techniques like cross-polarization optical coherence tomography or white-light scanning confocal profilometry [18]. In particular clinical indices have been subject of discussion [19]. The basic challenge is illustrated in a recent study by Rius-Bonet et al. [20] examining single clinical signs: the clinical signs with greatest balance between the sensitivity and specificity ‘convex areas flatten’ and ‘dull surface’ reach only a sensitivity of 63% and 47%, respectively, and a specificity of 71% and 89%, respectively. To date, however, no instrument-based procedure has been established that can reliably diagnose exposed dentin. Visual diagnosis, therefore, remains an important, universally applicable and inexpensive diagnostic procedure, but one that is fraught with difficulties.

The complexity of diagnosing erosive tooth wear and the involvement of 61 raters with heterogenous background, and the use of 49 areas varying in difficulty to come to a decision makes the original study a nice example to investigate the possibility to identify raters sharing a common notion with respect to the diagnostic problem of interest.

### Aim of the investigation

The aim of this investigation was to apply retrospectively a specific approach to identify clusters of raters with high similarity in their decisions and hence probably reflecting a common notion that exists among a particular cluster of raters. We further aim to illustrate how subsequent analyses of the clusters identified may allow us to obtain some insights into the notions, even if it is not possible to perform focus group interviews in raters sharing a common notion. Such interviews would be the obvious next step in case of a prospective study.

## Materials

**Fig. 1:**
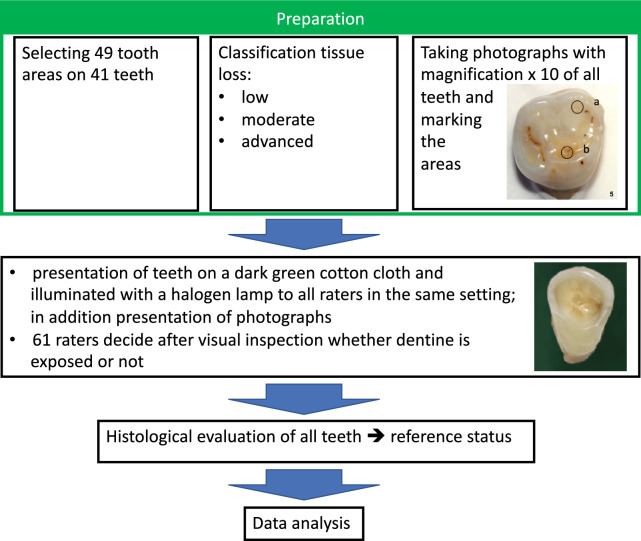
Workflow of the original study

This investigation is based on a study published in 2006 [9] and a further investigation in 2023 [21]. The original study [9] aimed to investigate the diagnostic accuracy of the visual distinction between “dentine unexposed” (negative status) and “dentine exposed” (positive status) including 61 raters. Forty-one teeth with signs of tooth wear were selected from a pool of extracted human teeth. The teeth were labelled with numbers from 1 to 41 according to the order of examination. One area was selected for examination in 33 teeth and two areas in 8 teeth, thus, there were 49 total areas for examination (33 + 16). The labelling of the areas corresponded to the tooth number, followed by the letters “a” or “b” in the case of two areas. A histological evaluation of the teeth as described in Ganss et al. [9], which was performed after finishing all ratings to ensure blinding, resulted in a number of only five areas with the status “dentine not exposed”. Hence, the status “dentine exposed” showed a prevalence of nearly 90%. The histological status acts as reference (true) status. Information on the patients providing the teeth was not available. A workflow of the study is shown in Fig. 1.

Twenty-three raters were scientists participating in an international scientific congress of the European Organisation for Caries Research. The other were dentists (*n* = 18) or students (*n* = 20) of the Dental Clinic, Justus Liebig University of Giessen. A booth was set up at the congress and every congress participant who was interested in the study and willing to diagnose the teeth was included. The participants from the clinic were then recruited in a similar way—there were no exclusion criteria except that the students had to be in the third year of study or higher, meaning that they had already seen patients and had also completed coursework on erosive tooth wear. The raters did not get any information about possible diagnostic criteria. There was only a short verbal instruction about the procedure. As the aim of the original study was to investigate the diagnostic accuracy in a real-world setting, the raters were not calibrated. In addition to the original teeth, the raters were given photographs (magnification × 10) of these teeth on which the area to be assessed was marked with a sticker. All raters were presented with the teeth in a standardized environment on a dark green cotton cloth illuminated with a halogen lamp. The raters had to mark on a paper sheet their selection between “dentine exposed” or “dentine not exposed” for each area. The teeth were presented in a fixed sequence (corresponding to the numbers used in this paper), and the raters had to make a decision before the next tooth was presented.

With respect to the raters, information on pre- or post-graduation, sex, age, professional experience, and specialization had been documented. As age was highly associated with graduation status and professional experience, it is ignored in the analyses. The two dichotomizations “students” vs “non-students” and “male “ vs “female” and within the non-students the dichotomizations “experience $\le$ 10 years” vs “experience > 10 years” and “specialized in erosion/cariology” vs “other specialization” were considered. Two further variables at the rater level were derived from the rater’s ratings. The first variable is the modified Youden index [22], which is defined by the average between sensitivity and specificity of the rater. Similar modifications have already been considered by Rücker and Schumacher [23] and Böhning et al. [24]. This variable reflects the diagnostic accuracy of the rater. The second is the rater-specific positive rate, i.e., the proportion of areas rated positive by the rater. Differences in the rater-specific positive rate between raters may reflect individual thresholds in the decision making.

**Fig. 2:**
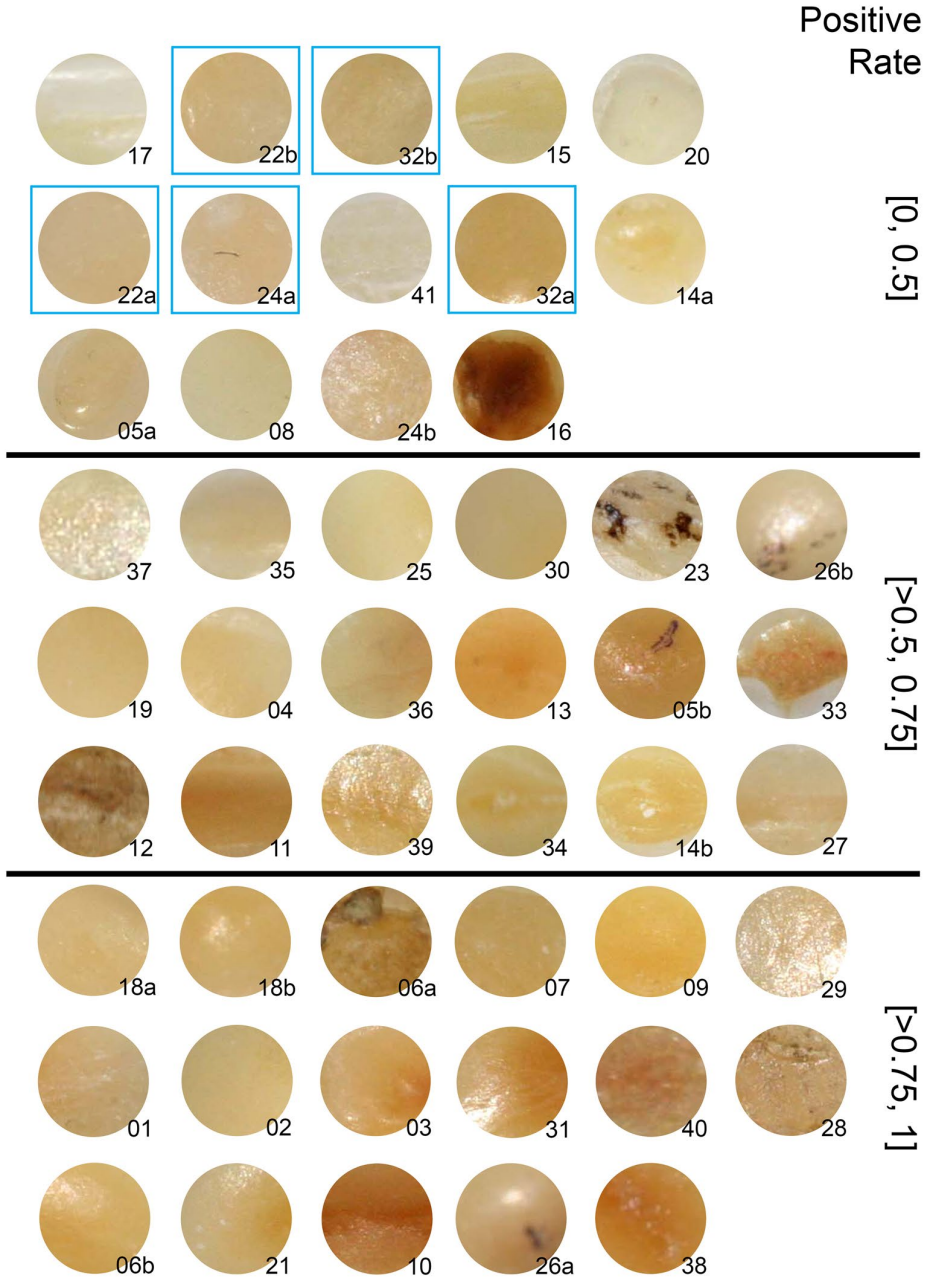
Images of the areas to be rated arranged according to increasing area-specific positive rate. Images are labelled with the area indicator. Blue frames indicate areas with the true status “dentine not exposed”

Two variables were considered at the area level. Prior to the assessment the principal investigator quantified the tissue loss of the areas according to the criteria by Ganss et al. [25] into minor, moderate or advanced. In a further investigation of the data Vach et al. [21] compared the area-specific positive rate (i.e., the proportion of raters giving a positive rating) with the images of the areas (Fig. 2) and identified this way another area-specific factor which influences the decision making: the background color of the area to be rated. In contrast to the rater-specific positive rate, which is a measure of the personal threshold values of the raters, the area-specific positive rate reflects how the area is perceived by the raters.

The specific feature of the background color could be covered by one single number, to which we refer as the “color index”. Details of the derivation of the color index can be found in Vach et al. [21].

## Methods

### Using the distribution of the area-specific positive rate to measure the share of a common notion

**Fig. 3:**
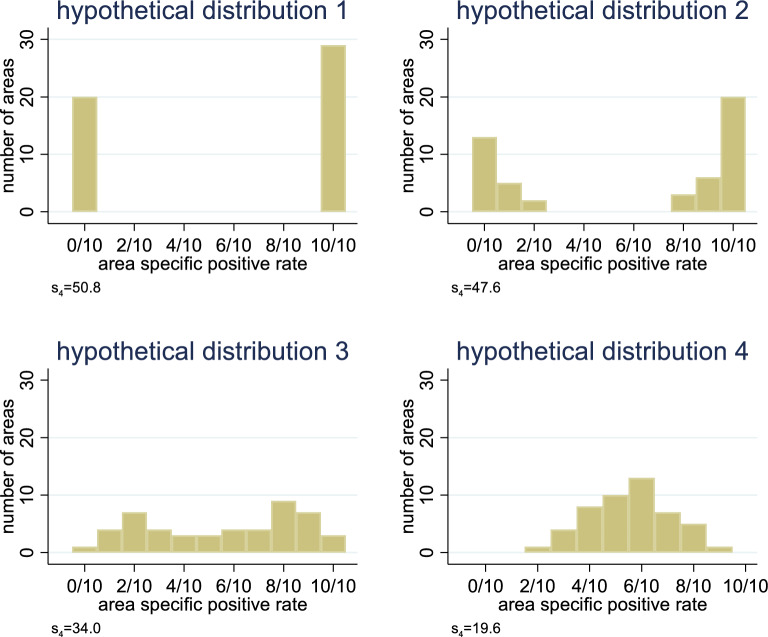
Hypothetical distributions of the area-specific positive rate in the 49 areas within a cluster of ten raters. The area-specific positive rate is expressed explicitly as a fraction

If raters share the same diagnostic notion, they should agree in many cases with respect to their rating. Hence, to assess whether a group of raters has a common perception, a simple approach is to examine the distribution of the 49 area-specific positive rates when these rates are calculated only for the raters in this cluster. For many areas, all raters should agree in their decisions on “dentine exposed” or “dentine not exposed”, i.e., all raters should give a positive or all a negative rating and the number of areas with conflicting ratings should be limited. On the other side, if a cluster of raters does not share any notion, then the rating of one rater is statistically independent from the rating of any other rater, and the distribution of the area-specific positive rate can be approximated by a Gaussian distribution. Figure 3 illustrates this with four different hypothetical distributions. It depicts the range of possible distributions of the area-specific positive rate for a cluster of ten raters rating the 49 areas of interest. In the first example a), all raters share exactly the same notion, such that for 20 areas they all give a negative rating (resulting in a positive rate of 0/10) and for 29 areas a positive rating (resulting in a positive rate of 10/10). In example b), the raters still have mainly the same notion and only rarely one or two raters deviate from the other raters. In example c), the picture is more mixed. There are still many areas with ten, nine or eight raters coming to the same positive or negative rating, but for some areas it seems to be more or less at chance level how the raters rate. In example d), the raters seem to lack any common notion, such that agreement among all of them never happens.

Figure 3 also illustrates the use of a simple measure for the degree to which the raters share common notions. This measure is denoted by *s*_*4*_ and it is a measure of the variation of the observed area-specific positive rates *p*_*a*_ (expressed as percentages). Similar to a standard deviation, it is based on considering the distances of the area-specific rates *p*_*a*_ from the mean rate $\overline{p }$ in $N$ areas:

$$s_{4} = \sqrt[4]{{\frac{1}{N} \mathop \sum \limits_{a} \left( { p_{a} - \overline{p}} \right)^{4} }}$$

By taking the fourth power (in contrast to taking the square as in a standard deviation), ${s}_{4}$ gives more weight to values far away from the mean rate and hence becomes in particular large if many areas are close to 0% or 100% with their area-specific positive rate.

### Systematic application of the measure

This measure was applied to the group of all raters as well as to eight subgroups, which could be built based on the four available dichotomous rater characteristics. This way first insights whether such subgroups may already share a common notion to a higher degree than the overall population of raters could be obtained.

However, the measure can be also used to look systematically for clusters of raters with a common notion by trying to maximize the value of ${s}_{4}$ across all possible subsets of raters. However, the number of possible subsets of the 61 raters is around 2.3 × 10^18^. Even if a computer would be able to check 1 million subsets in a second, it would still need 73,000 years to check all subsets. Hence, there was a need for a search strategy to determine subsets of interest, and the strategy used in this paper is outlined in S2 Appendix Search Strategy.

### Investigation of clusters of raters identified

Once clusters of raters sharing a common diagnostic notion have been identified this way, there are several ways to get some insights into the cluster-specific notions:

- Studying the composition of raters

Raters with the same notion may share certain characteristics, e.g., a long experience. Hence, the distribution of rater characteristics across the clusters was systematically compared.

- Pairwise agreement and disagreement of the area-specific positive rates

Notions can differ from one cluster to another in various ways. Some areas may still be perceived in the same manner, but some may change their role with respect to being perceived mainly as positive, mainly as negative, or in an ambiguous way. Such a change can be directly measured by a change in the area-specific positive rate, e.g., from 100% to 50%. To identify such differences across notions, the agreement or disagreement of area-specific positive rates for each pair of clusters was inspected in scatterplots.

- Areas with the widest span in positive rate

Areas with a distinct change in the perception from one notion to another can inform about cues interpreted differently by different notions. Consequently, the areas with the widest span in positive rate across the clusters and evaluated their characteristics including the visual appearance – similar as in Vach et al [21] were identified.

- Cluster-specific association of the area-specific positive rate with area-specific factors

Positive ratings may be triggered by different cues in different notions. Consequently, the cluster-specific degree of association of the area-specific positive rate with the two area-specific variables (tissue loss grading and color index) across the clusters were compared. The Spearman correlation was used as measure of association.

### Concordance of raters with the identified notions

Having identified a cluster of raters with a common notion, it remains the question to which degree some of the remaining raters may have shared at least partially the notion of the raters of this cluster. A simple measure for this is the concordance rate between the ratings of a single remaining rater and the area-specific positive rates of this cluster. The concordance rate is the relative frequency of area pairs for which the order of the ratings of the single rater coincides with the order given by the area-specific positive rate (neglecting the pairs with equal ratings or equal rates). The higher the concordance rate between a rater and a cluster, the more the rater may have shared the notion of the cluster. However, the rater may have used another threshold for decision making. In this case, the concordance rate can be high, but nevertheless the rater will not be included in the cluster when applying the search strategy. The distribution of the cluster-specific concordance rates in the remaining raters for each cluster was systematically investigated to get a further impression about the popularity of the notions identified. Finally, the characteristics of raters with a low concordance to all notions were inspected, i.e., those who may have a highly individual notion of “dentine exposed”.

### Summary of methodology

In summary, this investigation attempted to find clusters of raters with similar notions based on a measure of agreement among the raters and using a predefined search strategy. The identified clusters were analyzed with regard to various factors to uncover the similarities of the raters within a cluster and to identify potential cues used in decision making.

All computations were done with STATA (Version 17.0, College Station, TX, USA).

Five raters did not provide a rating for up to 6 areas, resulting overall in 13 missing ratings out of 2989 possible ratings. All measures based on ratings are based on the available ratings.

## Results

### Evidence for a common notion among all raters and in prespecified clusters of raters

**Fig. 4:**
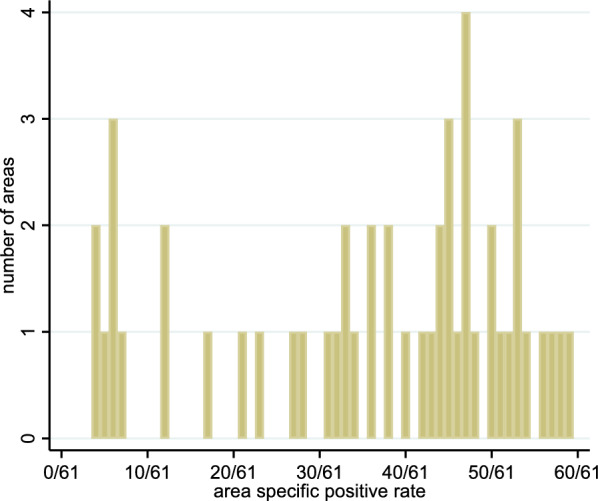
Distribution of the area-specific positive rate among all 61 raters

Figure 4 shows the distribution of the area-specific positive rates when considering all 61 raters. There are no areas for which all 61 raters agreed on the status of the area (i.e., a rate of 0/61 or 61/61). However, the distribution indicates that raters agreed to some degree for some areas. There were seven areas for which less than ten raters gave a positive rating (rate $\le$ 10/61) and there were ten areas for which less than ten raters gave a negative rating (rate $\ge$ 51/61). On the other side, there are 14 areas with rates between 21/61 and 40/61, indicating that the raters were far away from a common opinion about the status of the area.

**Fig. 5:**
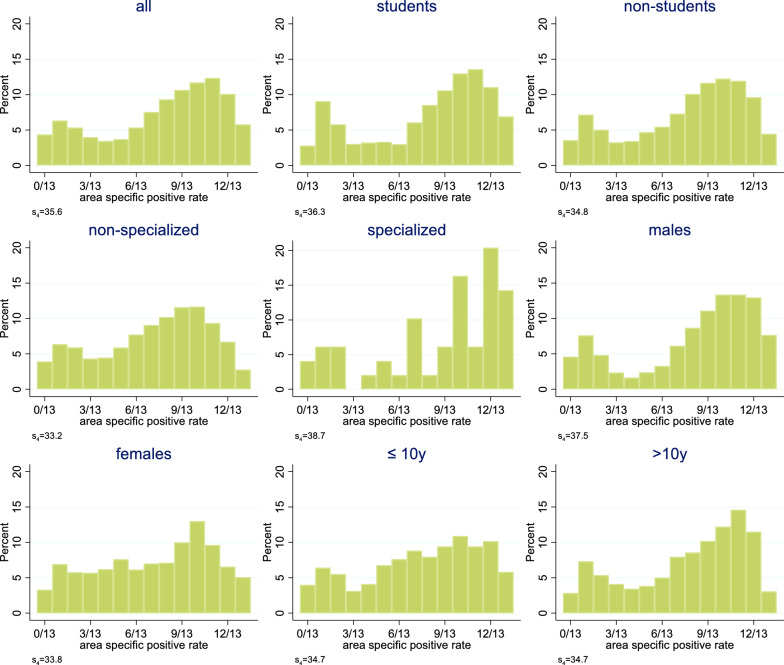
Distribution of the area-specific positive rate among all 61 raters and within eight clusters defined by rater characteristics. To allow a direct comparison among all nine analyses, the average distribution for randomly chosen subgroups of 13 raters are shown, cf. S1 Appendix Alignment

Figure 5 compares the distribution of the area-specific positive rate in all raters with the distribution in eight clusters defined by the four rater characteristics available. Slight differences in the tendencies to agree on many areas can be observed. In particular the specialized raters and the male raters tended to agree more often, i.e., showed higher values of ${s}_{4}$. Note that the tendency observed for males cannot be explained by the tendency observed for specialists, as the fraction of specialists was roughly equal among male and female non-students. Neither the raters with a high nor with a low experience seemed to have a notion of their own.

### Identification of clusters of raters with a common notion

**Fig. 6:**
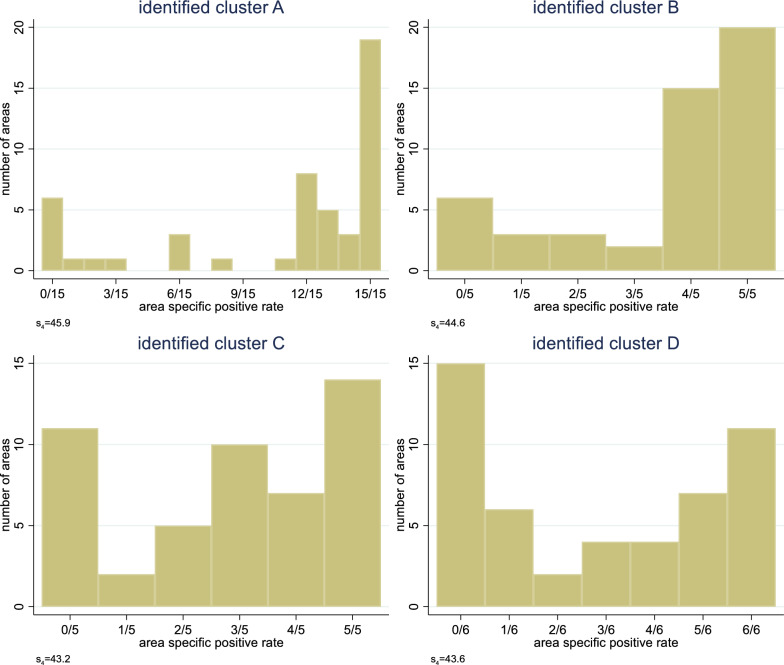
Distribution of the area-specific positive rate in four clusters resulting from the application of a systematic search strategy

The application of the systematic search strategy described in S2 Appendix Search Strategy resulted in four clusters with 15, 5, 5, and 6 raters, respectively. The essential property defining the clusters was their distribution of the area-specific positive rates in each cluster, which is shown in Fig. 6. The first cluster of 15 raters seemed to share rather stringently a common notion with 25 out of the 49 areas rated as positive by all raters or as negative by all raters. There were only five areas with rates between 4/15 and 11/15, i.e., a stronger disagreement among the raters. For the three other clusters, there were 25 or 26 areas with perfect agreement, which, however, is less impressive as the number of raters is only five or six. Similarly, there were more areas with disagreement among the raters. Hence, there is still an indication for a common notion within each cluster, but the agreement on this common notion is less pronounced.

### Composition of raters in the four clusters

Table 1 shows characteristics of the raters in each cluster. The raters in cluster A were characterized by a high diagnostic accuracy ranging from 0.82 up to 0.90, whereas the other clusters showed a distinctly lower accuracy with values only up to 0.85. In cluster D, the modified Youden index of 0.74 was below the average of 0.76 in all raters. Clusters A and B showed a similar distribution of the rater-specific positive rate with ranges from about 0.64 to about 0.81, whereas clusters C and D showed lower rates.

**Table 1:** Distribution of rater characteristics in the four clusters identified by the systematic search strategy

Statistics			Cluster A	Cluster B	Cluster C	Cluster D	All raters
N			15	5	5	6	61
Positive rate	mean		0.72	0.71	0.57	0.47	0.59
	range		0.65–0.82	0.63–0.81	0.51–0.61	0.43–0.51	0.12–0.90
Modified Youden index	mean		0.90	0.81	0.82	0.74	0.76
	range		0.82–0.95	0.72–0.85	0.78–0.84	0.64–0.78	0.49–0.95
Female	N		2	2	1	4	25
Male	N		13	3	4	2	36
Student	N		5	4	1	4	20
Non-Student	N		10	1	4	2	41
Specialized	N		5	1	3	0	13
Non-Specialized	N		5	0	1	2	28
Experience > 10y	N		6	1	4	0	21
Experience $\le$ 10y	N		4	0	0	2	20
Specialized	ex > 10y	N	3	1	3	0	9
	ex $\le$ 10y	N	2	0	0	0	4
Non-Specialized	ex > 10y	N	3	0	1	0	12
	ex $\le$ 10y	N	2	0	0	2	16

Please note that students have been excluded from the “Experience” and “Specialized” categories

Only in cluster D, there were more females than males. Cluster A was dominated by 13 males. It included five students, five specialists, and five non-specialists, and three of the specialists and three of the non-specialists had more than 10 years of experience. Clusters B and D were dominated by four students and cluster C was dominated by three specialists with more than 10 years of experience.

### Pairwise agreement and disagreement of area-specific positive rates between clusters

**Fig. 7:**
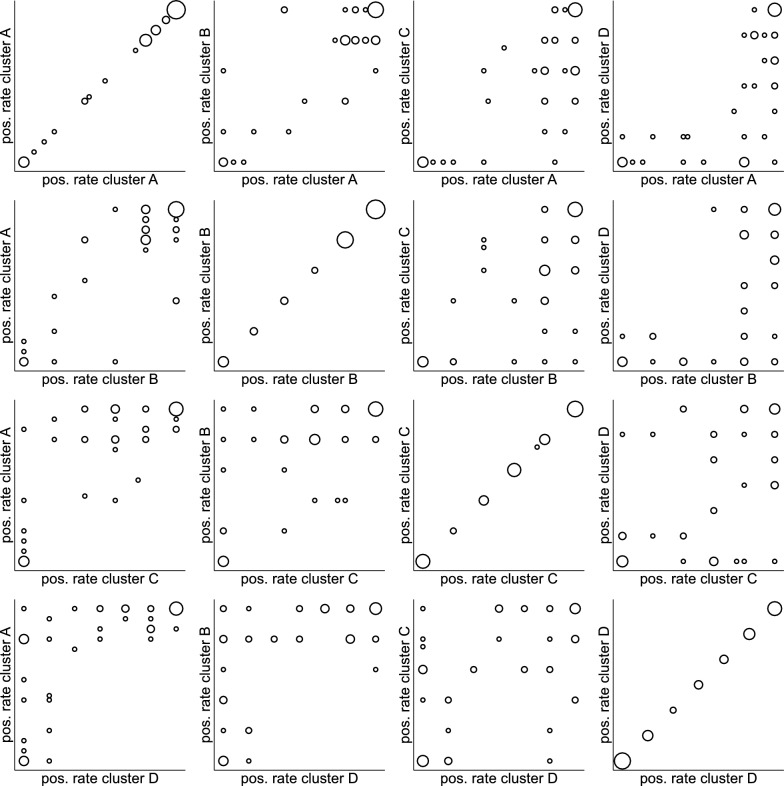
Pairwise agreement of area-specific positive rates between the four clusters. The size of the bubbles is proportional to the number of observations

Figure 7 depicts the pairwise agreement and disagreement of the area-specific positive rates across the four rater clusters. In each pairwise comparison, the bubbles in the lower left and upper right corner indicate that there was a substantial percentage of areas for which all raters from both clusters completely agreed on a positive rating or a negative rating, respectively. Clusters A and B showed only few areas with positive rates far away from the diagonal, i.e., differed distinctly in the positive rate. Cluster C differed more distinctly from cluster A and B with some areas with positive rates close to 1 in cluster A and B but very low rates in cluster C. The same type of difference at an even more pronounced level could be observed when comparing clusters A and B with D. When comparing clusters C and D, positive rates could both distinctly increase and distinctly decrease.

### Areas with a wide span in positive rate

Table 2 identifies the areas with the most pronounced differences in positive rates across the four clusters. Many different patterns with respect to the distribution of high and low positive rates across the four clusters could be observed. A common pattern observed in seven areas was a rate of 0% in cluster D, but positive rates of at least 80% in one other cluster and of at least 60% in two other clusters. All areas in Table 2 showed a positive true status. Only one of the ten areas had an advanced tissue loss grading, which coincides with the findings from Vach et al. [21] that a low degree of tissue loss makes the diagnosis more challenging. The color index showed a wide range. The visual inspection of the areas in Fig. 2 indicated that six of the areas identified in Table 2 showed acquired discolorations that could not be removed by tooth cleaning: 23, 37, 16, 26b, 5b, 24b. Such discolorations may have led to varying perceptions and consequently to differences in the positive rate across the clusters. However, the areas did not share the same pattern of positive rates across the four clusters, suggesting that the discolorations did not change the perception in a uniform way.

**Table 2:** Ten areas with a span (difference between maximal and minimal positive rate) of at least 0.8 across the four clusters

Area	Span	Cluster A	Cluster B	Cluster C	Cluster D	True status	Grading	Color index
23	0.80	0.80	0.80	0.60	0.00	+	Minor	0.50
37	0.80	0.80	0.80	0.40	0.00	+	Minor	0.44
35	0.80	0.80	0.80	0.60	0.00	+	Minor	0.52
16	0.80	0.80	0.40	0.60	0.00	+	Minor	0.91
19	0.80	0.80	0.40	0.80	0.00	+	Minor	0.65
26b	0.80	0.80	1.00	0.20	0.83	+	Advanced	0.55
5b	0.87	0.87	0.80	0.00	0.83	+	Moderate	0.81
8	1.00	0.40	1.00	0.60	0.00	+	Moderate	0.46
34	1.00	1.00	1.00	1.00	0.00	+	Moderate	0.67
24b	1.00	0.40	1.00	0.00	0.17	+	Moderate	0.59

### Cluster-specific association of the area-specific positive rate with area-specific factors

Table 3 depicts the correlation of the area-specific positive rate in each cluster with the color index and the tissue loss grading. A correlation with the color index was present in all clusters, but most pronounced in cluster D. The correlation with the tissue loss grading was much weaker in all clusters, but again most pronounced in cluster D.

**Table 3:** Correlation of the area-specific positive rate with the color index and the tissue loss grading of the areas within the four identified rater clusters

	Cluster A	Cluster B	Cluster C	Cluster D
Color	0.47	0.37	0.41	0.60
Grading	0.03	0.17	0.11	0.23

### The concordance of raters with the four identified notions

**Fig. 8:**
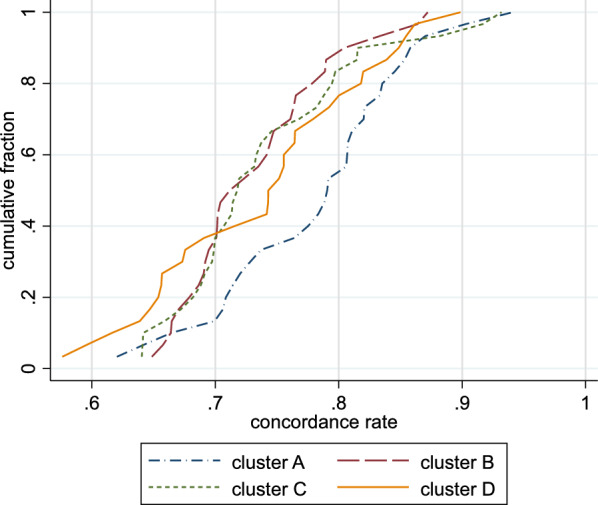
Distribution of the concordance rate between ratings of raters outside of a cluster and the average area-specific positive rate in the cluster for the four clusters identified

Figure 8 depicts the distributions of the concordance rates with respect to the four different clusters. For clusters A and C, there were raters outside of these clusters with a concordance rate above 0.9, whereas this was not the case for clusters B and D. The notion of cluster A seemed to be most popular also for other raters with nearly 45% of raters showing a concordance rate above 0.8, whereas this frequency was below 25% for all other clusters.

### Raters with a low concordance to all four notions

Among the overall 61 raters, 20 raters succeeded in reaching a concordance rate of 0.8 to all four clusters, suggesting that they all took into account the four notions identified to some degree. Among the 30 raters not attached to any of the four clusters, 18 reached a concordance rate of 0.8 to at least one of the four clusters. The remaining 12 raters with their characteristics are listed in Table 4. They seem to represent the whole spectrum of raters from experienced specialists over less experienced non-specialists to students and they cover males and females. The positive rate and the diagnostic accuracy were varying, too, but for most raters the accuracy was below the average modified Youden index of 0.76.

**Table 4:** 12 raters with a concordance rate of less than 0.8 to all four clusters

Maximal concordance-rate	Sex	Graduation status	Experience	Specialization	Positive rate	Modified Youden-index
0.67	f	Non-student	> 10 y	Other	0.33	0.68
0.70	f	Student			0.88	0.65
0.70	f	Non-student	$\le$ 10 y	erosion/cariology	0.51	0.67
0.71	m	Student			0.59	0.72
0.74	f	Student			0.39	0.49
0.75	m	Non-student	> 10 y	Other	0.63	0.85
0.76	f	Non-student	$\le$ 10 y	erosion/cariology	0.49	0.49
0.76	m	Non-student	> 10 y	erosion/cariology	0.80	0.50
0.77	m	student			0.45	0.75
0.78	m	Non-student	$\le$ 10 y	Other	0.55	0.70
0.78	f	Non-student	> 10 y	Other	0.65	0.53
0.79	m	Non-student	$\le$ 10 y	Other	0.55	0.81

### Summary of results

When looking systematically for clusters of raters we could identify one cluster (A) with a common notion shared by 15 raters. This notion seemed to be also rather successful, as all 15 members had a rather high diagnostic accuracy. Five members of this cluster have been specialized in erosion/cariology, but the cluster also included five students and five non-specialized non-students.

In additional, three smaller clusters could be identified pointing to three further shared notions of less popularity.

- The five raters of cluster B were in their ratings still rather close to cluster A, but their notion implied a loss in diagnostic accuracy. It was a notion mainly popular among students.
- The notion of cluster D was associated with an accuracy close to the average over all raters and a rater-specific positive rate close to 50%. Within this cluster, the area-specific positive rates correlated to a higher degree with the color index and the tissue loss grading than in the other clusters. This cluster may reflect raters who strongly believed that the prevalence of a positive status was close to 50% and who used the background color and the tissue loss in their decision making.
- The five raters of Cluster C did neither share the notion of cluster A, but in a very different way compared to cluster D. Three out of five members of this cluster were specialists, and four had an experience of at least 10 years. The accuracy was comparable to cluster B, but the positive rate was distinctly lower than in cluster A and B. Raters with a high diagnostic accuracy (above 0.8) could be found in three clusters, suggesting that different notions are compatible with a high accuracy. Indeed, there is no evidence that the best raters share a common notion: for the subset of the 15 raters with the highest Youden index the value of s_4_ is only 30.7, i.e., lower than in all pre-defined subsets considered in Fig. 5.

## Discussion

### Main findings

The present investigation aimed to illustrate how clusters of raters with a common notion can be identified and how identified clusters can be used to get further insights about the notion.

In a first step, the analysis of eight pre-specified subgroups of raters indicated that raters specialized in erosion/cariology appeared to be more likely to share a common notion about diagnosing erosive tooth wear than any other subgroup. However, they seem still to be rather heterogenous with many areas splitting the raters in two halves with a positive or a negative rating, respectively.

In a second step, a systematic search for clusters were performed and four clusters could be identified which may represent different notions. The clusters varied in their composition with respect to the three rater groups. Cluster A representing the most successful notion (with respect to diagnostic accuracy) included five specialists, five students and five non-specialized non-students varying in experience. This suggests that this notion is to some degree popular among specialists but does not necessary require much practical experience. The fact that some students already share this notion may reflect that they may have been influenced in developing their notion by some mentors or that the notion included rather intuitive elements, which are – however – taken up by only a minority of the students. Cluster D consisted of four students and two non-specialists with less than 10 years of experience, and it may hence reflect a popular notion about diagnosing erosive tooth wear for dentists with limited experience. Cluster C may represent a notion popular among experienced dentists, which were, however, not realizing the high prevalence of true positives in the study.

The four clusters covered only about half of all raters. Some of the raters not covered by any cluster showed nevertheless a high concordance with the notion of some clusters, with cluster A being again rather popular. However, some raters showed no concordance to any of the four notions. This indicates the existence of raters who have a highly individual notion not shared by many others. An individual notion does not seem to be associated with any rater characteristics.

The raters in cluster A with the most popular notion were dominated by males. Only in one cluster, the majority of users identified was female. This may indicate that female dentists tend to have a notion of their own. However, it remains miraculous how the males in cluster A could follow a common notion, as they cover different subgroups such as students, specialist or non-specialists. Moreover, among the 12 raters with least concordance to all four notions identified, we found the same number of males and females, indicating that also males can have a notion of their own.

This investigation underlines the challenge of understanding the role of the background color in diagnosing erosive tooth wear. As pointed out by Vach et al. [21], the existing literature indicates that the color of the dental tissue is not a good indicator for the exposure of dentin. However, the association with the color index observed in cluster A characterized by a very high accuracy suggests that it seems to be wise to look at some signs which are at least correlated with the color index. On the other side, the results for cluster D may indicate that it is not wise to by guided too much by the background color in diagnosing erosive tooth wear.

The role of visual signs in decision making was also corroborated by the result that six out of ten areas with large discrepancies in the positive rate across the four clusters showed acquired discolorations that could not be removed by tooth cleaning. This suggest that such discolorations were indeed regarded as a cue in decision making – in different ways in different clusters following different notions, – and did not lead to disagreement among raters sharing a common notion. In general, it is not implausible to use discolorations as a cue, as color pigments may be more likely to be embedded in dentine than in enamel.

It remains the question, why clinicians may have developed different notions. One potential explanation may be the existence of different phenotypes among patients suffering from dental erosion, as suggested by a recent study [26].

### Potential clinical impact

Since the original study was already performed more than 15 years ago, (and information on the identity of the raters was lost due to anonymization) it was not possible to contact the raters again. This has prevented the potentially most useful step: gathering the raters with a common notion in semi-structured focus interview sessions and finding out how their common notion can be described in conceptual and clinical terms. This would have opened the opportunity to learn more about good or poor cues used by the raters and may have allowed to develop corresponding guidelines and to optimize teaching and calibration procedures.

**Fig. 9:**
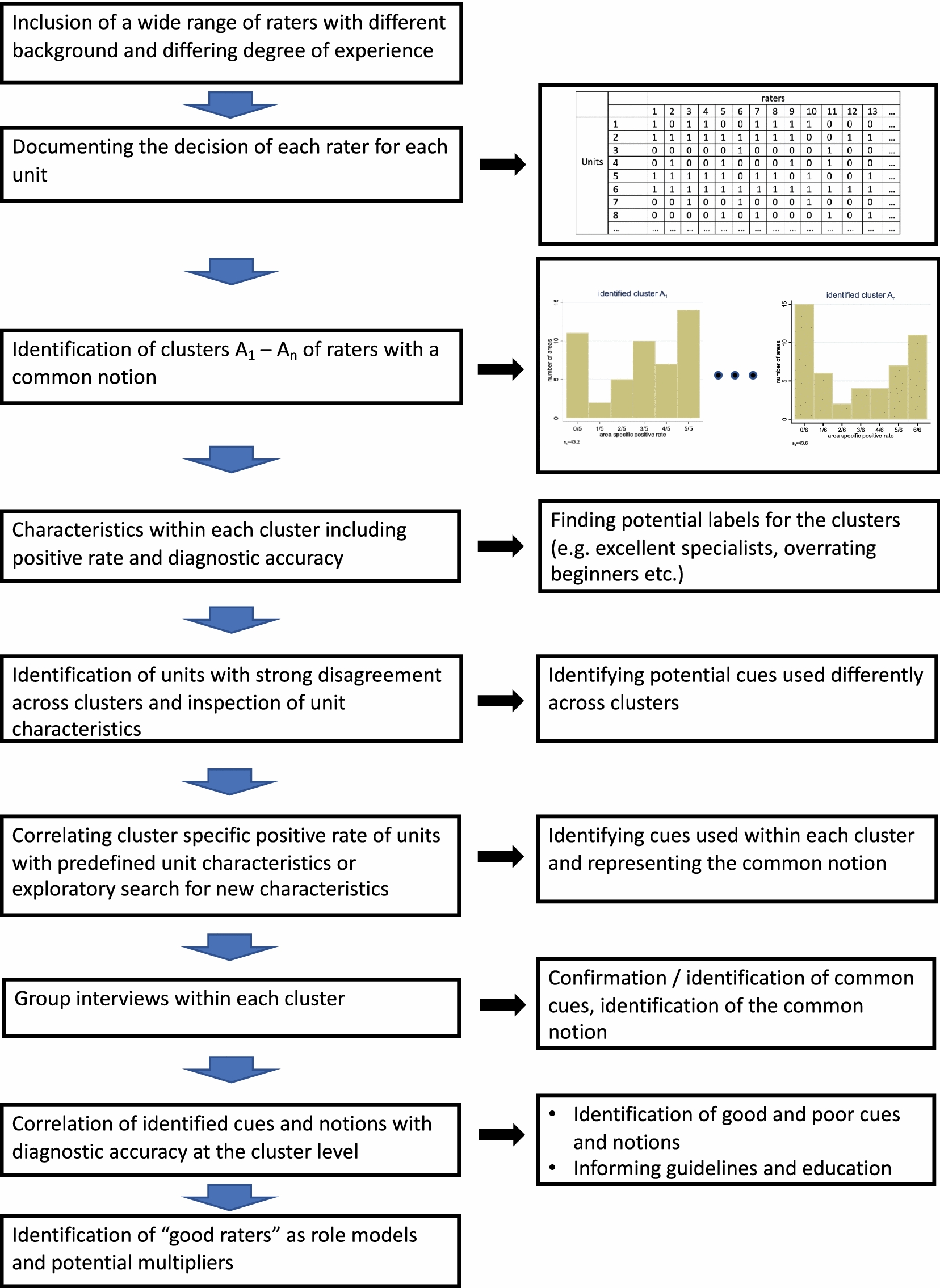
Workflow of potential application in future diagnostic studies

However, this step will be possible in future studies involving a sample of raters from a relevant population of raters, if analyses as those presented in this paper are part of the primary statistical analysis plan. As pointed out by Vach et al.[21], dentistry can be seen as a perfect field for this type of studies. Figure 9 summarizes the potential in future diagnostic studies.

As many works in the field of dentistry already deal with the use of AI as a diagnostic tool [27–30], it might be argued, that in future the application of artificial intelligence for imaging interpretation may overcome the issue of differing diagnostic notions varying across raters. Indeed, if all potential raters are replaced by the same AI tool, there is no longer any variation in notions. However, it is likely that we will have different AI tools, varying in the type of algorithm or the learning sample used to train the tools. Then it might be of interest to compare the “notions” of the different tools. The approach presented in this paper could then contribute to make AI tools more explainable.

### Methodological considerations

The search for clusters of raters with a common notion was approached in this paper by a systematic search strategy, aiming at maximizing a criterion chosen specifically to address the question of interest. The criterion used in this paper is closely related to the fourth central moment and the kurtosis, a measure of the deviation of the shape of a distribution form normality [31, 32]. However, preliminary experiments demonstrated, that ${s}_{4}$ is better suited to identify the clusters of interest than the kurtosis.

Results from such a systematic search should be interpreted with some caution. For any cluster identified, there may be many other clusters differing by one or two raters with very similar values of *s*_*4*_. The selection of one specific cluster is hence somewhat arbitrary. Moreover, it is hard to judge the statistical significance of the findings, i.e., whether the results are above chance level.

There are many other approaches to identify clusters of raters which show similarities in their decision making. For example, hierarchical clustering methods based on pairwise similarity measures can be used. S1 Figure shows a dendrogram achieved using Cohens’ kappa [33] as a similarity measure and applying the average linkage clustering technique [34]. For each of the four clusters A, B, C, and D some raters are already clustered together in this dendrogram, but not all of them. This underlines that a common notion means more than high pairwise agreement. Latent class analysis (LCA) is an another very popular approach [35]. However, when applied directly to our data set, this results in identifying clusters of raters with similar positive rate, but not necessarily high agreement. Adding the rater-specific positive rate as a covariate can avoid this effect, but in our experience, it still does not result in identifying clusters of raters with high values of ${s}_{4}$.

The high concordance rate of some raters outside of a cluster identified suggests that we may have overlooked some raters with very similar notion, which just have a different threshold in their decision making. Defining criteria allowing to catch also these subjects will be of interest. The diagnostic setting considered in this paper has to be distinguished from settings in which the signs and symptoms used as cues by the raters are known and measurable. Then it is possible to try to reconstruct the notion of each rater by relating the measured signs and symptoms to her or his decisions. For example, this can result in a diagnostic score for each rater giving weights to the single signs and symptoms, and variation in weights across raters allow identifying different strategies.

### Strengths and limitations

To our knowledge, this is the first investigation in a dental context that attempts to study the similarity in diagnostic decisions and tries to identify notions. This work can be also seen as a contribution to the discussion how to handle the general insight that the accuracy of a diagnostic test should not be regarded as a constant value [36–38]. This discussion covers both the variation across the raters as well as the variation across different subgroups of patients, varying in diagnostic difficulty. The latter aspect has already been addressed using the same data set [21]. The basic limitation of this investigation is the retrospective analysis of a data set generated many years ago. This prohibits conducting interviews with the raters. However, a workflow could be proposed to be applied in future, prospective applications.

## Conclusion

In diagnostic accuracy studies with multiple raters, it is possible to identify raters with similar diagnostic notions, in other words, individuals with similar underlying conceptual understanding that drives their diagnostic decisions. By interrogating clusters of raters with similar notions, we may have the opportunity to learn more about useful or useless cues in diagnostic decision-making. This would allow development or enhancement of guidelines on diagnostic decision-making.

## Supplementary Information

Supplementary Material 1

Supplementary Material 2

## Data Availability

The data generated or analyzed during this study are available as csv-file (S1 data) in the supplement.
