# Supplementary material for: Identifying clusters of raters with a common notion of diagnosing erosive tooth wear: a step towards improving the accuracy of diagnostic procedures
Source: Eur J Med Res. 2025 Jan 9;30:15. doi: 10.1186/s40001-024-02260-1 (PMC11716257; doi:10.1186/s40001-024-02260-1)

Supporting information

S1 Appendix Alignment: Making the measure s_4 comparable across clusters of different size.

When applying the measure s_4 to clusters of different size *N*, the results can be slightly misleading. The larger the cluster, the more unlikely it becomes that all raters agree for one specific area and that we can observe extreme results such as area-specific positive rates of exactly *0/N* or *N/N*. To address this issue, the following approach was used in Figure 4, aiming to apply the measure to nine different clusters: The smallest cluster size N_{min} = 13 over all clusters of interest was determined, from each cluster 1000 random samples of size 13 were drawn, and the average distribution of the area-specific positive rate was used for the visualization and the computation of s_4.

S2 Appendix Search Strategy: A search strategy to identify clusters of raters with a common notion.

A common notion can already be detected at the level of pairs of raters: If two raters agree on nearly all areas, this is an indication for a common notion. Consequently, a rather natural search strategy is to start with all pairs of raters with some evidence for a common notion, and then to try to find triples, quadruples etc by adding a rater and selecting those clusters with high values of the target function, ie, the value of s_4 for the cluster.

However, there are some challenges to implementing such a strategy:

- Since there are some areas with a high positive rate and some areas with a very low positive rate when considering all raters, it has to be expected that we observe signs for a common notion for all pairs.
- The values of the target function are affected by random fluctuations in the data. With increasing size of the clusters, the number of possible clusters increases, and the maximal value achieved within clusters of equal size reflects to an increasing degree the random noise. Hence, comparisons of the value of the target function across clusters of different size should be avoided.
- If there is an optimal cluster of raters of size N and there is another optimal cluster of size $N + 2$ which can be constructed by adding two raters, there is no guarantee that the two subsets of size *N+1* which can be built by adding one rater are among the optimal ones at size *N+1*.
- If there exist for one size an optimal subset, there will be many other subsets similar to this subset differing by one or a few raters and with a similar value of s_4. Since we were interested in finding different subsets of raters with distinctly different notions, it is important to ensure that one subset is not dominating the search strategy.

To address these issues, the following search strategy was used. The starting point are all pairs of raters defining the candidate subsets of size 2. Candidate subsets with more than two raters are then constructed using the following recursive algorithm:

- From all subsets of size $N - 1$ candidate subsets of size N are constructed by adding one rater.
- A subset can generate maximally K new subsets, ie, only the K new subsets with maximal value of the target function are kept.

- From the remaining subsets of size N all subsets are removed, for which there exists more than K subsets with a higher value of the target function and only differing by one rater.

The resulting list of candidate subsets was systematically searched for pairs of non-overlapping subsets. Within pairs of subsets with the same overall size (ie, the sum of the sizes of the two subsets), the pair with maximal value of the sum of the values of the target function weighted by the subset size was selected. These values tend to decrease with increasing size, but a rather distinct decrease was observed when reaching a size of 21. This way, the clusters A and D were identified.

We extended the previous step by looking for triples of non-overlapping subsets. However, this resulted only in a suggestion to split the cluster A with 15 raters into two subsets and adding some raters. Taking into account that the 15 raters already defined a cluster with a very clear common notion, this new solution was not regarded as useful.

Finally, the whole search strategy was repeated excluding the raters of cluster A and D identified so far. This resulted in the two clusters B and C with five raters.

In the search strategy, the value of K was set to 3 for $N \geq 4$. Only for $N = 3$ the value 5 was used.

S1 Fig: The pairwise similarity across raters expressed by Cohens' kappa and visualized by a dendrogram based on application of the average linkage clustering technique. Letters indicate raters from the identified clusters A-D.

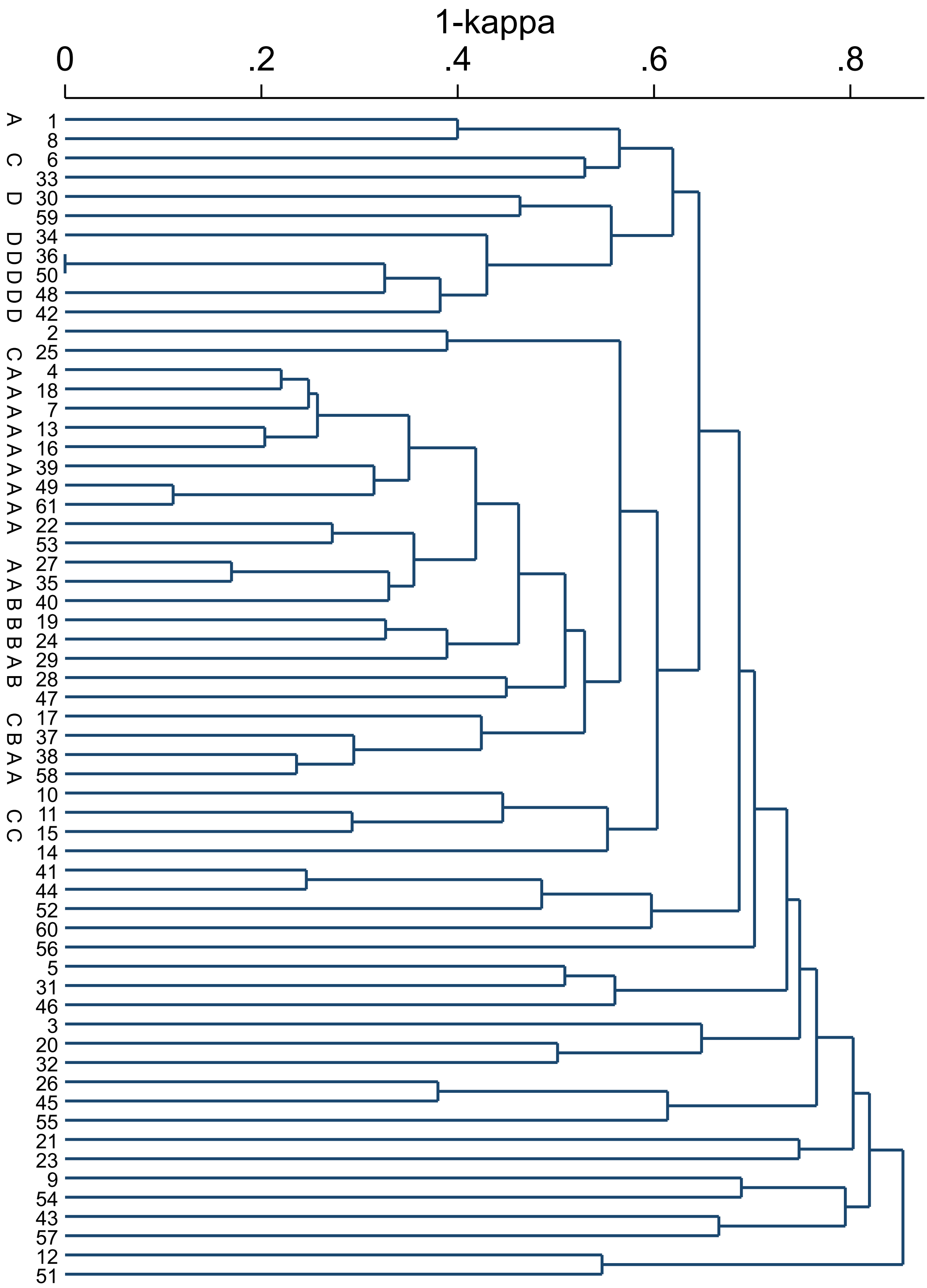

Supplement: Supplementary file 2 — Supplementary Material 2 [file 40001_2024_2260_MOESM2_ESM.pdf]
